# Stable Cobalt-Mediated Monolithic Dye-Sensitized Solar Cells by Full Glass Encapsulation

**DOI:** 10.1021/acsaem.2c00765

**Published:** 2022-05-26

**Authors:** Fátima Santos, Jorge Martins, Jeffrey Capitão, Seyedali Emami, Dzmitry Ivanou, Adélio Mendes

**Affiliations:** † LEPABE—Laboratory for Process Engineering, Environment, Biotechnology and Energy, Faculty of Engineering, University of Porto, Rua Dr. Roberto Frias, 4200-465 Porto, Portugal; ‡ ALiCE—Associate Laboratory in Chemical Engineering, Faculty of Engineering, University of Porto, Rua Dr. Roberto Frias, 4200-465 Porto, Portugal

**Keywords:** dye-sensitized solar cells, monolithic, cobalt redox electrolyte, stability, temperature, hermetic encapsulation, indoor photovoltaics

## Abstract

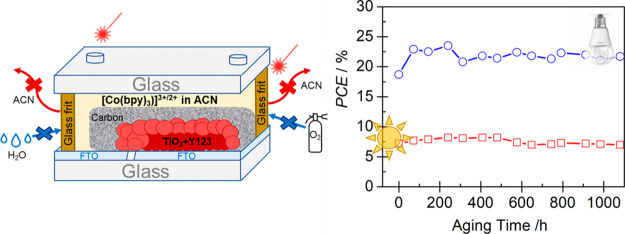

Dye-sensitized solar cells (DSSCs) emerged in the market as one of the most promising indoor photovoltaic technologies to address the need for wireless powering of low-consuming electronics and sensor nodes of the internet of things (IoT). The monolithic design structure of the cell (M-DSSCs) makes the devices simpler and cheaper, and it is straightforward for constructing in-series modules. The most efficient DSSCs reported so far are Co(III/II)-mediated liquid junction cells with acetonitrile electrolytes; however, they are mostly unstable. This study reports on highly stable cobalt-mediated M-DSSCs, passing thermal cycling tests up to 85 °C according to ISOS standard protocols. Under 1000 h of aging in the dark and under simulated solar and artificial light soaking, all tested cells improved or retained their initial power conversion efficiency. Advanced long-term stability was achieved by eliminating the extrinsic factors of degradation, such as the interaction of the cell components with the environment and electrolyte leakage. This was obtained by encapsulation of the devices using a glass-frit sealant, including the holes for filling up the liquid components of the cells. The hermeticity of the encapsulation complies with the MIL-STD-883 standard fine helium gas leakage test, and its hermeticity remained unchanged after humidity–freeze cycles according to IEC 61646. The elimination of extrinsic degradation factors allowed reliable assessment of inner factors accountable for aging. The impact of the ISOS-protocol test conditions on the intrinsic device stability and long-term photovoltaic history of the M-DSSCs is discussed.

## 1 Introduction

Dye-sensitized solar cells (DSSCs) are the third generation photovoltaic (PV) devices^1^ that recently reached a certified power conversion efficiency (PCE) of 13%.^2^ Since their inception in 1991,^3^ DSSCs have attracted vast research interest due to their low manufacturing cost and sustainability,^4,5^ outstanding PCE under dim and artificial light,^1,6^ and aesthetics.^1^ These features turned DSSCs feasible to produce PV glazing for buildings^7,8^ and for agrivoltaic applications.^9^ With the emergence of the internet of things (IoT) and indoor low-power consuming wireless communication devices, the development of energy sources for powering them emerged imperative.^10,11^ DSSCs are among the most promising indoor PV technologies to address this challenge:^12,13^ they display a record PCE of 34.5% under indoor illumination,^2^ do not contain toxic lead nor tin, can be produced in flexible substrates, and can be semitransparent with true colors for eye-pleasant interior integration;^1,6^ DSSCs for indoor light conversion have recently entered the PV market.^1,13^

The most efficient state-of-the-art DSSC devices use Co(III/II) or Cu(II/I) complexes as redox shuttles.^2,6,14−16^ Copper-mediated devices hit certified 1 sun and artificial light conversion records,^1,2^ while cobalt DSSCs achieved a reported PCE above 14% under AM1.5G light.^15,16^ The best performing cobalt cells are liquid junctions with volatile acetonitrile (ACN) electrolytes.^15−17^ Cobalt electrolytes are transparent compared to the copper counterparts, which allow producing efficient bifacial devices^18,19^ for gaining *ca.* 20–40% of output power;^20,21^ this is relevant for indoor use where the maximum power obtained from a single-face cell hardly reaches tenths of a milliwatt per cm^2^.^11^

Since the advent of cobalt DSSCs, their stability has been questioned^22^ and tremendous research efforts have been made for identifying the deterioration mechanisms.^23−35^ Strategies for more stable devices include tuning the chemical structure of the cobalt complexes,^23−25^ adjusting concentration of Co(III) and Co(II) species,^26,27^ decreasing volatility of the solvents,^28,29^ selecting proper electrolyte additives,^23,26,29,30^ identifying stable counter-electrodes,^27,31^ and developing of quasi-solid-state gel-electrolyte devices.^32−35^ The long-term stability of cobalt DSSCs loaded with ACN or with other less volatile electrolytes is always challenging due to the inevitable leakage of the solvent through the device’s sealed perimeter.^27−29,31^ Most of the time, the sealant used is a Surlyn thermoplastic that has poor barrier properties, low softening point temperature of *ca.* 93 °C, and displays poor mechanical stability under temperature cycling tests.^36^ Thermal cycling or damp heat tests, desirable for PV product certification, was successfully applied in iodide DSSCs.^37,38^ However, to our knowledge, the same tests have never been reported for cobalt DSSCs with ACN electrolytes, even at a minimum setpoint temperature of 65 °C, as recommended by ISOS protocols.^39^ Electrolyte volatility also limits light-soaking tests for tracking PCE history; only a few studies consider testing periods for more than 1000 h.^26,30^ Gao et al.^26,30^ studied the effect of electrolyte compositions and additives based on 1000 h aging tests under simulated solar light and 60 °C. The authors reported that a higher concentration of Co(III/II) complexes favors the device’s stability. 4-*tert*-Butylpyridine (TBP)-free electrolytes allowed one to fabricate a device with an initial PCE of 0.7%, which improved over the testing period up to 3.5%. A deterioration of *ca.* 15 and 8% in an initial PCE of *ca.* 6% was observed with and without LiClO_4_ additives, respectively; the low photostability in the presence of Li^+^ ions was assigned to the fast dye degradation and electron lifetime decrease. Kamppinen et al.^27^ studied the stability of poly(3,4-ethylenedioxythiophene) (PEDOT) and Pt counter-electrodes by exposing the devices to 1 sun light soaking for 2000 h at 40 °C. Counter electrodes made from Pt allowed a more stable device; however, the losses of PCE were 26 and 53% after 1000 and 2000 h, respectively. Jiang et al.^28^ observed *ca.* 34% of PCE drop in the best ACN device after 2000 h of light soaking at the open-circuit potential and 20 °C, also reporting issues with electrolyte leakage. Hybrid redox couples, combining cobalt complexes with ionic liquids, have been developed, showing good stability under ambient conditions in the dark over 120 days; an increase of 10% in the PCE was observed in the devices with TBP additives.^40^

Both intrinsic and extrinsic factors are affecting the stability of a PV cell.^41^ The extrinsic factors are related to the encapsulation hermeticity. However, when the barrier properties of the encapsulant are unknown, it is impossible to clearly distinguish whether the intrinsic or extrinsic factors are responsible for the degradation of the device.^41^ Surprisingly, the extrinsic stability of cobalt DSSCs has never been addressed, although practically all the studies report electrolyte leaks.

Encouraged by the ability of cobalt-DSSCs to deliver high PCEs under sunlight and artificial illumination,^42^ we report the most stable cobalt-DSSC device (M-DSSC) obtained by robust and exceptionally hermetic glass encapsulation. The device edges and the electrolyte injection holes were glass-sealed using a low-temperature laser-assisted process.^43−45^ The seal tightness fully complies with the MIL-STD-883 standard, helium leakage test, with no signs of deterioration after passing the humidity–freeze cycle test according to the IEC 61646 standard. After eliminating the extrinsic factors of degradation, we focused on the effect of the standard ISOS test conditions on the charge transfer at the interfaces of M-DSSCs, dye desorption, redistribution of the cobalt ions in the working device, and their impact on stability and overall PCE.

For the first time, glass-encapsulated M-DSSCs with an ACN-based cobalt electrolyte and the organic dye Y123 successfully passed several ISOS tests, including thermal cycling up to 85 °C, 1000 h of shelf-aging, and 1000 h of solar and artificial light soaking with a passive load, displaying the most steady history of PV metrics and an average PCE of 8% under sunlight and 22% under artificial illumination.

## 2 Experimental Section

### 2.1 Reagents and Materials

Titanium diisopropoxide bis(acetylacetonate), acetylacetone, anhydrous isopropyl alcohol, 4-*tert*-butylpyridine (TBP), LiClO_4_ (99.90%), and anhydrous acetonitrile (ref. 271004, 99.80%) were purchased from Sigma-Aldrich and used without additional purification. Screen-printable TiO_2_ pastes (30NR-D, WER2-O) and FTO-coated glasses (7 Ω/sq) were from GreatCell Solar. Screen-printable glass paste to produce glass frit for the device sealing was based on Bi and Zn oxides. The thermoplastic sealant (Meltonix 1170–60, 60 μm Surlyn) and the screen-printable graphite/carbon-black paste (Elcocarb B/SP) were acquired from Solaronix. Complexes Co(III/II)tris(bipyridyl)tetracyanoborate (Eversolar Co-300 and Co-200) were from Everlight and Y123 organic sensitizer was ordered from Dyenamo. Titanium tetrachloride (99.90%) and *t*-butanol (99.5%) were purchased from Acros Organics.

### 2.2 Fabrication of Fully Hermetic M-DSSCs

**Figure 1:**
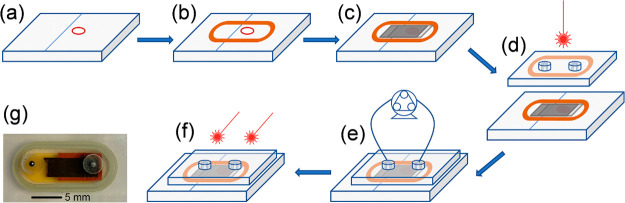
Manufacturing flow chart (a–f) and photograph (g) of glass-sealed M-DSSCs.

Fully hermetic M-DSSCs were produced using laser-assisted glass-frit encapsulation of the cell edge,^43−45^ and the electrolyte injection holes were sealed with glass. Figure 1 illustrates the sequence of the device assembly procedures.

Scribed and cleaned FTO-coated glasses were covered with *ca.* 80 ± 5 nm blocking layer of TiO_2_ deposited by spray pyrolysis at 450 °C; the spray solution was made of 7.0 mL anhydrous isopropyl alcohol + 0.6 mL titanium diisopropoxide bis(acetylacetonate) + 0.4 mL of acetylacetone. A circular shaped (4 mm in diameter) mesoporous TiO_2_ layer with a thickness of 7 ± 1 μm was formed by screen printing of 30NR-D titania paste and annealing at 500 °C (1 h). The mesoporous TiO_2_ layer was treated by dipping in the 40 mM TiCl_4_ solution at 70 °C for 30 min, dried, and annealed at 500 °C (Figure 1a). A thin oval-shaped frame of the glass paste was screen printed on the FTO glass, around the mesoporous TiO_2_ layer, and sintered at 490 °C for 60 min (Figure 1b). An insulating spacer layer, with a thickness of 6 ± 1 μm, was deposited atop mesoporous TiO_2_ by screen printing of conventional rutile paste (WER2-O) followed by sintering at 500 °C for 1 h. A 20 ± 5 μm thick graphite/carbon-black counter electrode was applied over the insulating spacer layer by doctor blading of Elcocarb B/SP paste and sintered at 420 °C for 45 min (Figure 1c). The cover glass was prepared by engraving the electrolyte injection holes in thin capillaries fabricated by laser ablation on one side of the glass (Figure S1) and depositing an identical glass frit sealing frame on the other side. The front FTO glass was aligned with the cover glass so that the frit frames match each other (Figure 1d); a laser-assisted glass-frit bonding process, as described elsewhere,^43^ was used for sealing the cell edges. The photoanode sensitization was then obtained by closed-loop pumping for 5 h (recirculation) of 10 mL, 0.1 mM Y123 dye in a solvent acetonitrile/*t*-butanol (1:1) volume ratio (Figure 1e). The cavity of the cell was then cleaned through passing acetonitrile and dried with nitrogen. Electrolyte made of 0.165 M Co(II) and 0.045 M Co(III) tris(bipyridyl)tetracyanoborate complexes, 1.2 M TBP, and 0.1 M LiClO_4_ was injected into the cavity; the tops of the injection glass capillaries were melted by fast heating using the laser beam (Figure 1f), completing the device assembly and glass encapsulation (Figure 1g).

For comparison with glass-sealed M-DSSCs, a batch of Surlyn-sealed devices was also prepared. The cover glass with pre-drilled cylindrical injection holes (0.7 mm) was bonded to the device using a Surlyn spacer and hot pressing. After electrolyte injection, the holes were closed using Surlyn films with lamella glass on top.

Hermeticity and robustness of the device encapsulation were assessed by the standard helium gas leak test MIL-STD-883 method 1014.10^46^ before and after five humidity–freeze cycles corresponding to the IEC 61646 standard.^47^ Each humidity–freeze cycle included sample exposure to 85 °C and 85% relative humidity for 20 h, then cooling to −40 °C, without humidity control, and holding at −40 °C for 30 min. Interested readers may consult ref (48) for details of the He-leakage and humidity–freeze test conditions.

Briefly, the obtained He-gas leak rates allowed the calculation of the equivalent air leak rates (*L*). *L* is used to assess the hermeticity of the device. Values of *L* below the reject limit indicate that the device hermeticity meets the standard. Considering the internal volume of the sealed devices is 1.2 × 10^–2^ cm^3^, the reject limit (*L*) is 1 × 10^–7^ atm·cm^3^·s^–1^.^46^

**Figure 2:**
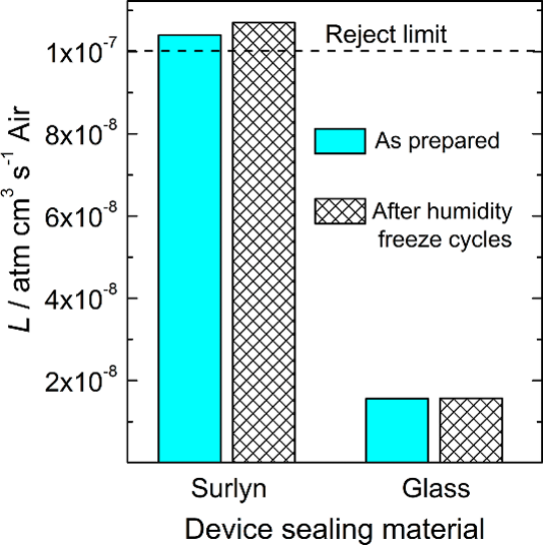
Leak rates *L* according to MIL-STD-883 test conditions of glass- and polymer-sealed empty cells before and after five humidity–freeze cycles according to the IEC 61646 protocol.

Figure 2 presents leak rates *L* for as-prepared empty devices sealed with glass and polymers, subjected to five humidity–freeze cycles.

Thermoplastic-sealed devices show *L* values above the reject limit, meaning poor hermeticity, which, in addition, degraded under humidity–freeze cycles. Glass-sealed cells display *L* rates *ca.* 10 times less than the reject limit, demonstrating superior hermeticity. Encapsulation with glass withstands the standard IEC61646 humidity-freeze test, showing no signs of hermeticity deterioration.

Glass encapsulation involves more manufacturing steps, where the new steps may affect the PCE of the devices. However, freshly prepared glass and polymer-sealed cells show almost identical current–voltage (*J*–*V*) characteristics (Figure S2). The average 1 sun PCEs of a batch of 7 devices were 8.0 ± 0.5 and 7.9 ± 0.4% for glass and polymer-encapsulated cells, respectively.

### 2.3 Characterization

A batch of 4 devices was subjected to (a) He leak rates, measured using an Adixen ASM 142 mass spectrometer with a sensitivity of 5 × 10^–12^ atm·cm^3^·s^–1^ to He and (b) humidity–freeze cycle test, performed in a climatic chamber—Fitoclima (Aralab). Detailed protocols of ISOS tests can be found elsewhere.^39^ For temperature effect studies, the temperature of M-DSSCs was controlled using an in-house made experimental setup^49^ constructed from a Peltier element (Marlow Industries, model RC12-6) connected to a Keithley power supply (model 2425C). An Autolab electrochemical station (PGSTAT 302 N, Metrohm) was used to record *J*–*V* characteristics and to perform the electrochemical impedance spectroscopy (EIS) characterization. The EIS data were analyzed in ZView software. A solar simulator MiniSol (LSH-7320, Newport) was used as a source of AM1.5G (100 mW·cm^–2^) light.

The stability of the glass-sealed and polymer-sealed M-DSSC devices was evaluated during 1000 h under continuous 850 W·m^–2^ light soaking at 45 °C in a climatic chamber Atlas SUNTEST equipped with a Xe lamp and AM1.5G filter; UV filter was used to cut the radiation below 390 nm.

A LED lamp (Osram, Class A+, 60 W, 2700 K with emission spectra presented in Figure S3) was used as the light source for the artificial light-soaking test. The incident light intensity from the LED lamp was calibrated using a radiometer Delta Ohm HD 2102.2.

During light-soaking tests, each M-DSSC was connected to a variable resistance as the passive load (Figure S4). The resistance was adjusted to keep the M-DSSCs at their nominal operating potential, that is, at the maximum power point. Adjustments of the connected resistance were made throughout the entire testing period, each time after measuring the device photoresponse.

The concentration of cobalt ions in the electrolyte before and after the device aging test was determined by dissolving a droplet (2 μL) of the electrolyte in 7 mL of distilled water and analyzing the solution using ICP-OES apparatus—iCAP 7000 (Thermo Scientific) with a detection limit for cobalt of 5 ppb.

## 3 Results and Discussion

### 3.1 PV Behavior of Cobalt M-DSSCs at the Reversible Heat Impact according to ISOS-T-1

The certification of a PV device typically requires thermal cycling in the temperature range exceeding working conditions. Glass-encapsulated M-DSSCs successfully passed one heat cycle from room temperature to 85 °C, according to the ISOS-T-1 protocol,^39^ showing no signs of electrolyte loss and retaining the initial PCE. Such a high setpoint temperature was never used before for testing devices with ACN because the boiling temperature of ACN is 82 °C. The Surlyn-based devices did not pass the test due to severe electrolyte leakage above *ca.* 67 °C.

**Figure 3:**
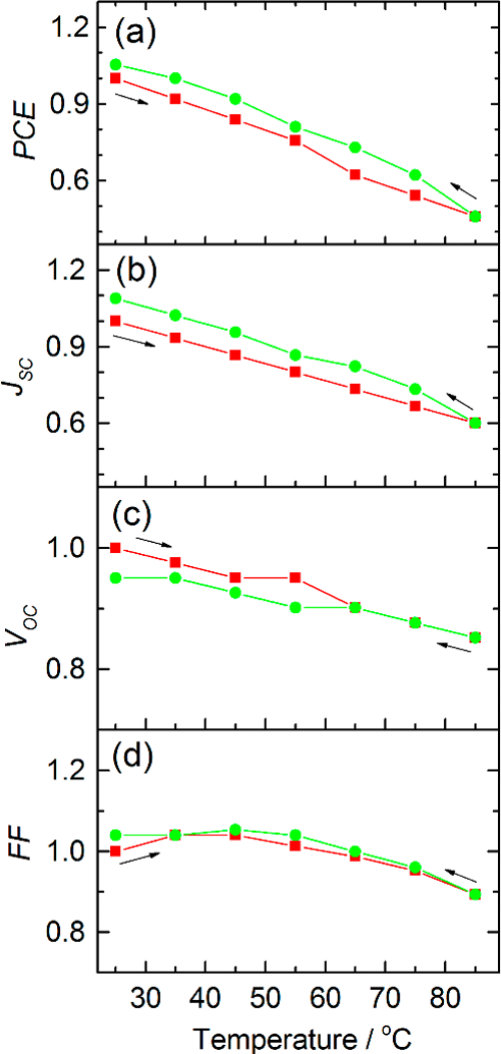
PV metrics of the M-DSSCs during the ISOS-T-1 temperature cycle 25 °C → 85 °C → 25 °C; 0.7 °C·min^–1^. The PCE (a), *J*_SC_ (b), *V*_OC_ (c), and FF (d) are normalized to the values obtained at the beginning of the cycle at 25 °C (Initial 1 sun PCE of 8.0%). The red squares and the green circles correspond to the rise and fall in temperature, respectively.

Figure 3 shows the normalized PV parameters of a glass-sealed M-DSSC at stepwise heating up to 85 °C, followed by a gradual temperature decrease.

The M-DSSCs display an expectable deterioration of the PCE with the temperature (Figure 3a), when moving from 25 to 85 °C, assigned to an increase of recombination at the photoanode/electrolyte interface.^49,50^ At 85 °C, the *V*_OC_ and FF remained *ca.* 90% of their initial values at 25 °C. The temperature accelerates the overall charge-transfer kinetics, including recombination, which may justify the decrease of *V*_OC_. The drop of the overall PCE is caused mainly because of *J*_SC_ decrease (Figure 3b); at 85 °C, the devices display 59 and 60% of the initial PCE and *J*_SC_, respectively. With the device cooling down, the *J*_SC_ and PCE slightly improved, increasing *ca.* 1.05 times after the device cooled down to 25 °C. However, the *V*_OC_ of the heat-treated devices decreased 5% from the initial value.

**Figure 4:**
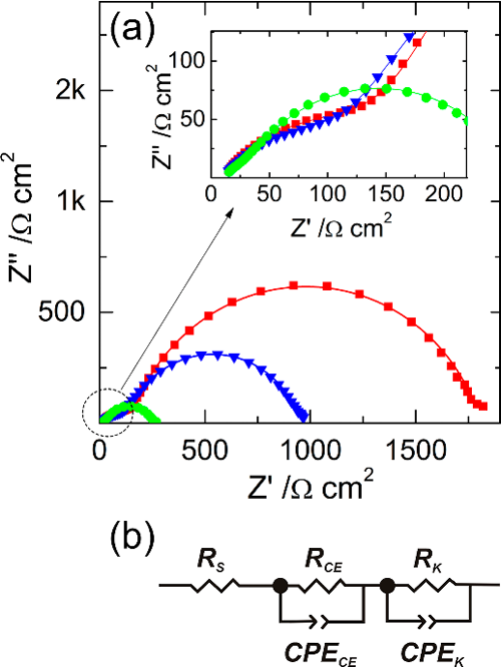
(a) Nyquist plots of the M-DSSC: initial at 25 °C—red squares; at 85 °C—green circles; after colling down to 25 °C—blue triangles. Solid lines stand for the fits to the equivalent circuit,^51,52^ shown in chart (b).

EIS measurements at *V*_OC_ (forward bias in the dark) accessed the changes in the device after thermal cycling (Figure 4). Impedance responses were collected before and after exposing the devices to the heating cycle and at the highest setpoint temperature of 85 °C.

The obtained Nyquist plots are typical for M-DSSCs^51,52^ and show two well-resolved time constants. The first one appears at the high-frequency range with low impedance (inset in Figure 4a) and corresponds to charge transfer at the counter electrode/electrolyte interface. Another time constant shows up at lower frequencies due to the charge transfer at the photoanode/electrolyte interface and is commonly used to estimate the rate of back electron recombination with electrolytes; higher resistance corresponds to less recombination.^53^ The equivalent electrical circuit shown in Figure 4b fitted well with the EIS response and it was used to identify the internal resistances of the devices. The elements in the model are series resistance (*R*_S_); charge-transfer resistances at the interface counter-electrode/electrolyte (*R*_CE_) and photoanode/electrolyte (*R*_K_); CPE_CE_ and CPE_K_ are the respective constant phase elements connected in parallel to the resistance. The values of resistances obtained from the fitting model are listed in Table 1.

**Table 1:** Values *R*_S_, *R*_CE_, and *R*_K_ (in Ω·cm^2^) of the M-DSSC at Different Temperatures

*T*/°C	*R*_S_	*R*_CE_	*R*_K_
initial 25 °C	10.0	148.7	1651.0
85 °C	10.6	22.4	227.4
final 25 °C	10.3	125.3	817.3

The series resistance of the devices was not affected by temperature and remained almost constant during the temperature cycling. *R*_CE_ decreased drastically with the temperature, being *ca.* 6.6 times lower at 85 °C when compared to the initial value—the charge transfer is accelerated with the temperature. When the device was cooled down, the *R*_CE_ did not return to its initial value—*ca.* 125 (Ω·cm^2^) at the end versus 148 (Ω·cm^2^) initial. This was assigned to the partial desorption of the dye from the carbon surface, which leads to more surface area of the electrode becoming accessible to electron exchange with cobalt ions. Although after the sensitization the cells were thoroughly cleaned at room temperature with a solvent, it is expected that a fraction of the dye did not desorb from the carbon layer. To confirm this assumption, a fresh carbon counter electrode (0.7 cm^2^) layer was deposited onto glass, immersed into Y123 solution, vigorously rinsed with acetonitrile at room temperature, and dipped into 1 mL of acetonitrile in a closed vessel. The vessel was held at 65 °C and at 75 °C for 10 min; after each temperature exposure, the absorption spectra of the acetonitrile were taken (Figure S5). The dye release from the carbon counter electrode is evidenced by an increase in the absorption in the 400–530 nm region, characteristic of Y123 dye.^54^

**Figure 5:**
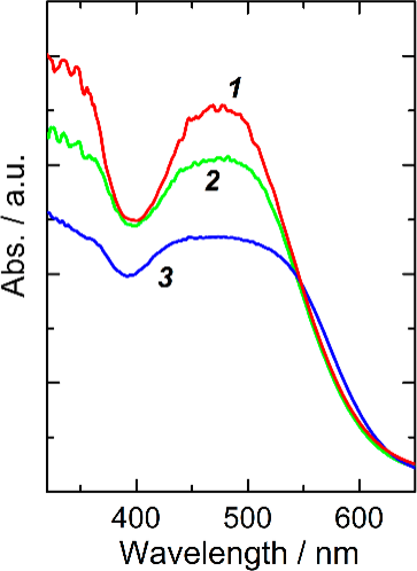
Absorbance spectra of the photoanode right after sensitization (1) and after being in contact with electrolyte in the device at 65 °C (2) and at 85 °C (3).

The resistance *R*_K_ is most strongly and irreversibly affected by temperatures. At 85 °C, *R*_K_ falls *ca.* 7.3 times, leading to faster recombination and to the drop of the photocurrent (Figure 3b). When the device is cooled back to 25 °C, *R*_K_ restored *ca.* 2.6 part of the initial—817 (Ω·cm^2^) at the end versus 1651 (Ω·cm^2^) initial. This is in line with previously reported electron lifetime reduction in heat-treated cobalt DSSCs^29^ and consistent with the observed drop in *V*_OC_ (Figure 3c). The slight improvement of the photocurrent (Figure 3b) was assigned to the positive shift of the conduction band edge potential of TiO_2_ due to dye desorption.^29^ However, strong dye desorption should cause the photocurrent to drop. The absorption spectra of the photoanode were taken before and after the immersion in a heated electrolyte (Figure 5).

Freshly sensitized photoanode displays a broad band centered at 438 nm, matching Y123 absorption. After being in contact with electrolytes at elevated temperatures, the absorption of the photoanode in the wavelength range of 400–530 nm drops *ca.* 1.2 and 1.5 times at 65 and 85 °C, respectively. The dye equivalent to the absorption decrease was desorbed from the mesoporous titania. Because the dye desorption does not lead to a significant photocurrent drop (the amount of the desorbed dye is rather high); on the contrary, it slightly improves, it can be assumed that adsorbed dye aggregates present at the mesoporous TiO_2_ surface of the fresh device leave the photoanode after the temperature cycle. A decrease in the *R*_K_ value after the heating cycle (Table 1) corresponds to this assumption. In the as-prepared device, that is, before the heating cycle, the aggregated dye molecules prevent charge transfer at the photoanode/electrolyte interface, originating a high *R*_K_ resistance. Hence, optimization of the sensitization conditions should be addressed in future studies.

### 3.2 Aging of Cobalt M-DSSCs in the Dark and under Simulated Solar Light Soaking according to ISOS-D-1 and Modified ISOS-L-2 Protocols, Respectively

**Figure 6:**
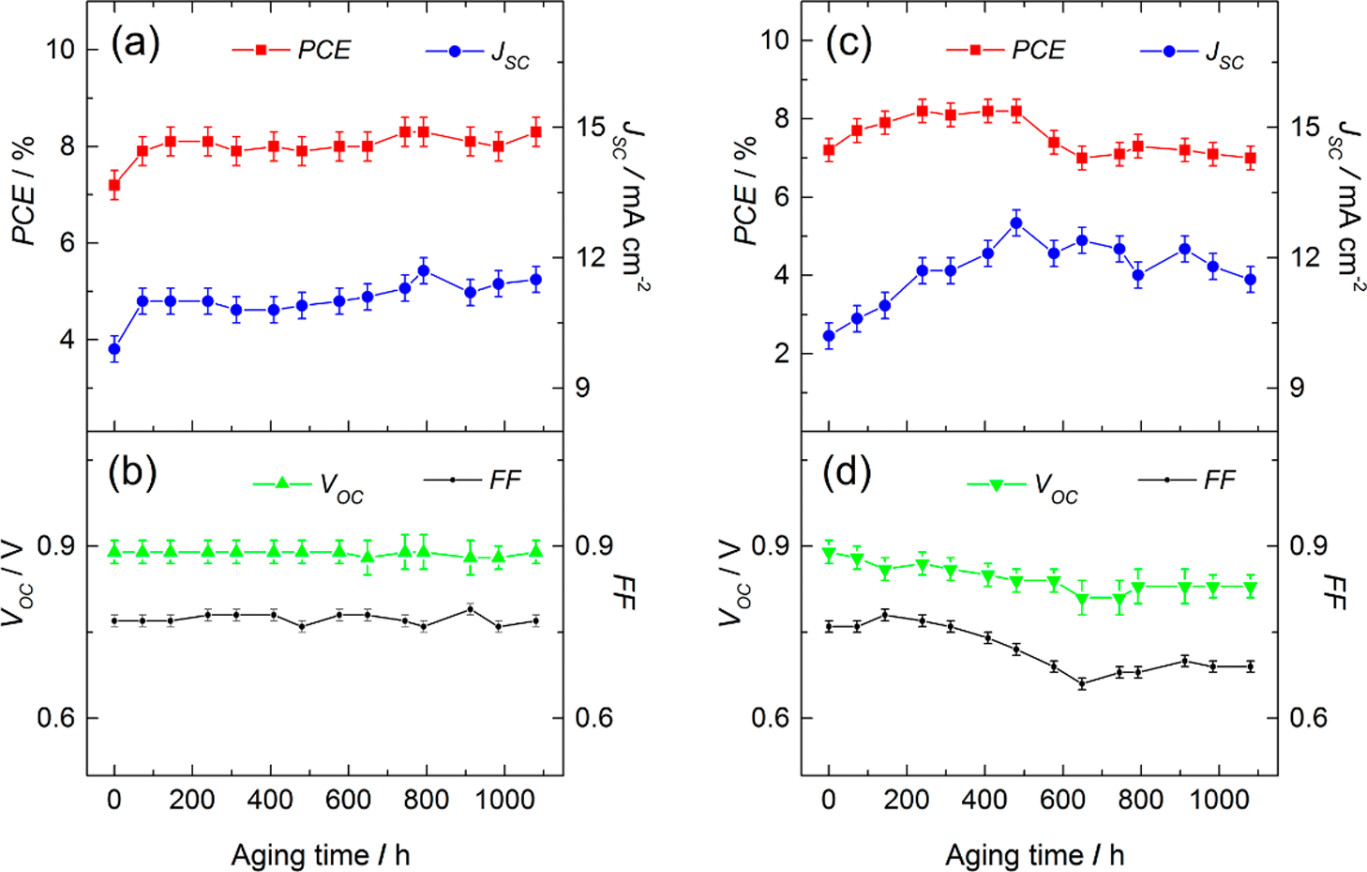
History of the PV metrics of hermetically sealed cobalt M-DSSCs during aging in the dark (a,b) and under simulated solar light soaking (c,d) according to ISOS-D-1 and modified ISOS-L-2 for 45 °C of operation.

Figure 6a,b shows the history of PV metrics of the glass-sealed M-DSSCs with natural aging under dark conditions according to ISOS-D-1.

**Figure 7:**
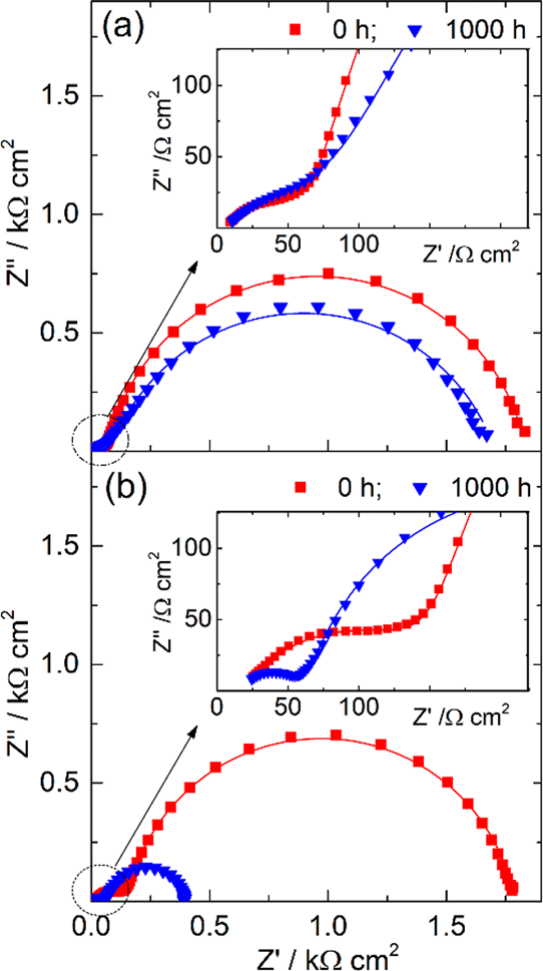
Nyquist plots of the glass-encapsulated M-DSSCs before (red squares) and at the end (blue triangles) of aging in the dark (a) and under simulated solar light soaking (b) according to ISOS-D-1 and modified ISOS-L-2 (45 °C), respectively. Solid lines are the fittings obtained using the electrical equivalent circuit shown in Figure 4b.

The cells display a well-defined increase of the PCE from *ca.* 7% initial to 8% within the first 200 h of aging and maintained it until the end of the 1000 h test, displaying full stability. Initial improvement of the PCE is mainly due to *J*_SC_, as FF and *V*_OC_ remained constant within the entire testing period. The EIS study revealed minor changes in impedance response of the cells before and after dark aging (Figure 7a). The charge-transfer resistance on photoanode *R*_K_ dropped slightly from 1.76 to 1.61 kΩ·cm^2^ after aging, while *R*_CE_ increased from 68 to 88 Ω·cm^2^. Small changes in the charge-transfer resistances point to the high stability of all internal components and interfaces of the M-DSSCs, which are in line with the history of the PV metrics (Figure 6a,b).

**Figure 8:**
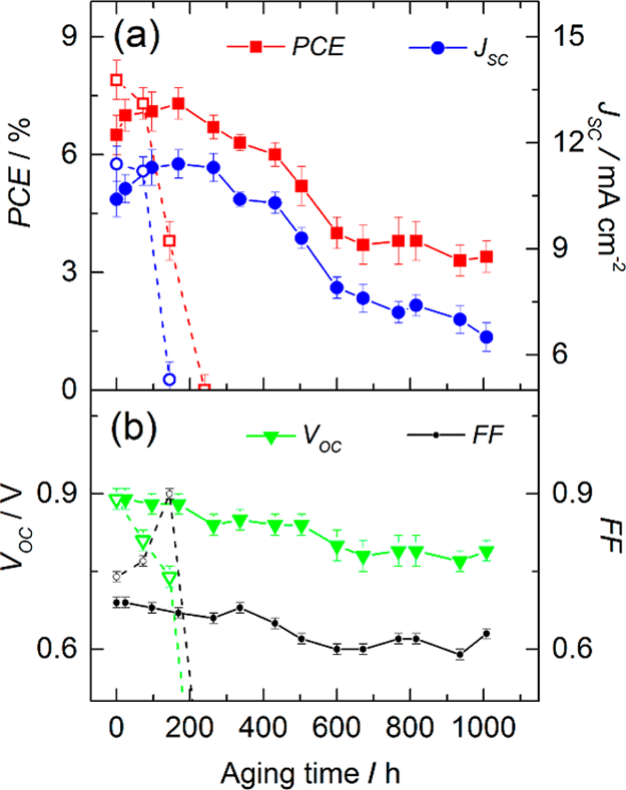
PV metrics versus time of the polymer-sealed M-DSSCs—PCE and *J*_SC_ (a), *V*_OC_ and FF (b) in the course of natural aging in the dark (solid symbols) and under simulated solar light soaking (empty symbols).

The batch of the devices sealed with Surlyn barely completed the ISOS-D-1 test, initially demonstrating a similar PCE improvement compared with the glass-encapsulated devices, within 200 h (Figure 8a).

However, after the initial 200 h, the degradation of the cell onsets, ultimately leading to the devices with only 3% of the PCE. The apparent reason for PCE deterioration was electrolyte leakage visualized at *ca.* 300 h. Nevertheless, devices with electrolyte leakage accomplished 1000 h of aging in the dark, constantly displaying a drop on *J*_SC_, *V*_OC_, and FF (Figure 8b). At the light-soaking test, devices encapsulated with Surlyn survived only 200 h until the PCE dropped to zero (Figure 8a).

Figure 6c,d shows PV metrics versus time of the glass-sealed M-DSSC as they were exposed to simulated sunlight (850 W·m^–2^) at *ca.* 45 °C and connected to an electrical resistance for operating at maximum power point conditions. From *ca.* 7.2% of the initial value, the PCE decreased after 1000 h of continuous illumination to 7.0%, which is within the error of the reads. The average response history of the glass-encapsulated devices was more complex. PCEs gradually increased until instant 200 h, reaching *ca.* 8.2%, stabilized for the next 300 h, decreased for the next 200 h, and stabilized afterward; *J*_SC_ constantly raised until instant 500 h and slightly decreasing afterward; and FF and *V*_OC_ displayed a slight dropping trend within a whole testing period of 1000 h.

The prolonged period of PCE constant improvement, 200 h, is observed for the first time; previous reports concerning the initial 500 h of light-soaking history of similar Surlyn-sealed devices display quite the opposite behavior.^26−28,30^ Gao et al.^26^ observed *ca.* 20% of PCE deterioration mainly due to the pronounced drop of *V*_OC_ and FF (1 sun; 60 °C); Jiang et al.^28^ reported very stable *V*_OC_, but descending FF and *J*_SC_ leads to *ca.* 25% loss of the original PCE (1 sun, 20 °C, open-circuit); and Kamppinen et al.^27^ obtained *ca.* 25% PCE drop caused by *V*_OC_ and FF decrease (1 sun, 40 °C, open-circuit).

The observed 200 h PCE improvement in continuously working devices is due to the continuous *J*_SC_ increase. The origin and mechanism of light-induced photocurrent improvement are quite interesting and profoundly studied elsewhere in ref (55) using LEG1 and PD2 as modeling dyes. Briefly, in the presence of a Lewis base such as TBP, adsorbed dye molecules are reorganized on the TiO_2_ surface and partially substituted with TBP. This process is accelerated under illumination and leads to an upshift of the dye LUMO level and downshift of the TiO_2_ conduction band; the driving force for electron injection is increased.

**Figure 9:**
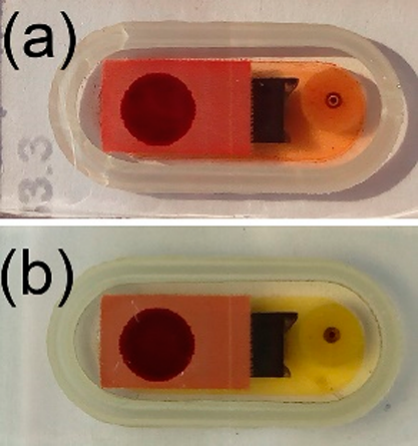
Photographs of the glass-sealed M-DSSCs taken before (*a*) and after 1000 h (b) of accelerated aging under 850 W·m^–2^ simulated solar light.

Later, after *ca.* 500 h, the photocurrent started to decrease, along with FF and *V*_OC_. The decrease of the FF value is assumed to be related to the increase of the concentration of Co(III) in the pores of the TiO_2_ mesoporous layer, which impairs the diffusion of Co(III).^28^ Accumulation of cobalt ions in the mesoporous layer should affect the electrolyte composition. Cobalt ion concentration in the electrolyte of aged devices has not been previously determined. Our findings show that the concentration of cobalt ions [total Co(III) and Co(II)] in the electrolyte surrounding the device, after light soaking, is 0.12 M, that is, became 1.7 times lower than the initial 0.21 M. This implies that cobalt complexes’ concentration drastically increases in the porous triple-layer sandwich of the M-DSSC. The exact determination of the amount of Co(III) and Co(II) mainly adsorbed by each layer of the monolithic device is challenging but should be addressed in future studies. The drop of the cobalt concentration justifies the bleaching of the electrolyte around the M-DSSC layers (Figure 9).

Impedance spectra of light-aged M-DSSCs display a drastic acceleration of charge transfer (recombination) at the photoanode/electrolyte interface: *R*_K_ decreases from 1.63 kΩ·cm^2^ to 337 Ω·cm^2^ at the end of the test (Figure 7b). The reduction of *R*_K_ fits well with the observed accumulation of cobalt ions in the M-DSSC layers; upon increasing cobalt complex concentration, the interfacial charge transfer increases, as expected. Among other reasons that could cause *R*_K_ to decrease is partial dye desorption.^55^ What is quite interesting here is that, despite the observed significant changes in the interfacial charge transfer, accompanied by changes of local concentration of cobalt complexes in the device, the overall PCE is practically not affected.

The PV metrics of the cells for the last *ca.* 250 h of the test is relatively stable, especially regarding *V*_OC_ and FF, and the full lifespan of the device cannot be predicted yet. A testing period longer than 1000 h is desirable for future studies.

### 3.3 Artificial 600 and 1000 lx Light Soaking

The requirements for long-term stability testing of PV devices under artificial indoor light are not clearly defined yet, and there is no standard lamp spectrum for indoor PV testing.^56,57^ As the share of LEDs in the market for indoor lighting is increasing, we used a LED lamp of Class A+ with a color temperature of 2700 K and emission spectra, as presented in Figure S3.

**Figure 10:**
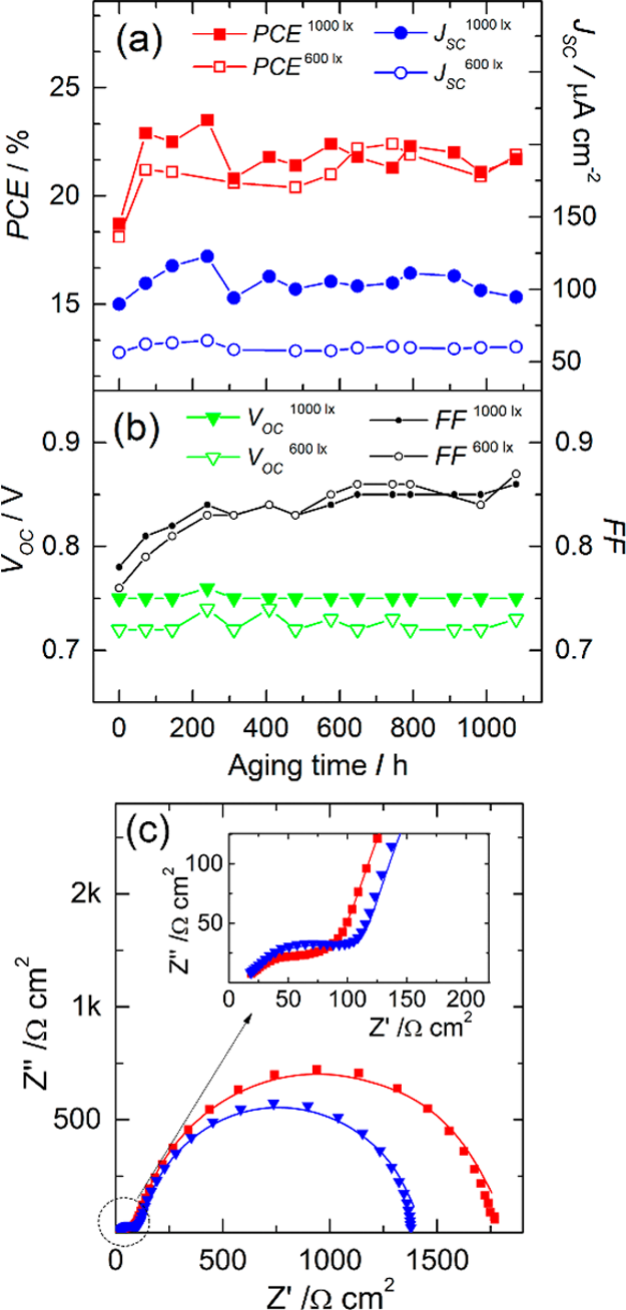
PV metrics vs time of the glass-sealed M-DSSCs in the course of 600 lx LED light soaking (a,b) and EIS responses of the devices before (red squares) and after (blue triangles) artificial light soaking test (c).

The devices were aged for 1000 h under 600 lx, a temperature of *ca.* 22–26 °C in a closed compartment, with a device connected to passive resistance load. *J*–*V* characteristics were recorded periodically under 600 and 1000 lx; Figure 10a,b presents the history of the PV metrics.

After 1000 h of aging, the overall PCE improved 20 and 16% under 600 and 1000 lx, respectively. The variations in PCEs are mostly related to small fluctuations in photocurrent, which might be due to minor variations in the incident light intensity. Aging under low artificial lighting indoors causes much less stress on cells, as shown in Figure 10c—EIS probing before and after the test. The charge-transfer resistance at the photoanode and counter electrode slightly decrease, similar to shelf-aging at room temperature in the dark (Figure 7a). To the best of our knowledge, this is the most stable DSSC employing an ACN cobalt electrolyte reported so far, under artificial light soaking. The history of PCEs shows that liquid-junction M-DSSCs are very promising as power sources, albeit with low but steady power under indoor illumination.

## 4 Conclusions

Hermetic encapsulation of liquid-junction cobalt M-DSSCs with ACN is absolutely essential for a stable PV response under ISOS testing conditions. Glass-encapsulated M-DSSCs showed fully stable PCE in the dark and under continuous operation under artificial light. Robust encapsulation allowed for the first time ever to test the devices with an ACN electrolyte according to the ISOS thermal cycle protocol at 85 °C. After heating to 85 °C, the M-DSSCs retained the initial PCE, however, with profound intrinsic changes attributed to dye desorption and acceleration of charge transport at the photoanode/electrolyte interface. Continuous 1000 h operating with a passive load under simulated solar light led to a drastic increase of charge transfer at the photoanode and redistribution of cobalt ions over the device; nevertheless, and for the first time ever, the PCE showed mostly a constant value over the entire testing period. This is one of the most impressive conclusions of this report; after increasing interfacial charge transfer, dye desorption, and concentration gradient of cobalt ions over the cell cavity, the overall PCE only slightly deteriorated or even remained unaffected.

Full glass encapsulation proved to be a keystone for the stability of cobalt-based DSSC devices and critical for an in-depth study of the internal degradation mechanisms. Longer tests are now possible, opening the doors to the development of more stable and performing devices.

## Supporting Information Available

Photograph of the capillary structure of injection holes, *J*–*V* curves of M-DSSCs, emission spectra of LED lamp, typical photograph of the M-DSSC for ISOS-L2 testing, and absorbance spectra (PDF)
