# Supplementary material for: Stable Cobalt-Mediated Monolithic Dye-Sensitized Solar Cells by Full Glass Encapsulation
Source: ACS Appl Energy Mater. 2022 May 26;5(6):7220–9. doi: 10.1021/acsaem.2c00765 (PMC9773422; doi:10.1021/acsaem.2c00765)
Supplement: Supplementary file 1 — ae2c00765_si_001.pdf [file ae2c00765_si_001.pdf]

# SUPPORTING INFORMATION

## Stable cobalt-mediated monolithic dye-sensitized solar cells by cell full glass encapsulation

Fátima Santos,[1,2] Jorge Martins,[1,2] Jeffrey Capitão,[1,2] Seyedali Emami,[1,2] Dzmitry Ivanou,[1,2,*] Adélio Mendes[1,2,*]

*1. LEPABE - Laboratory for Process Engineering, Environment, Biotechnology and Energy, Faculty of Engineering, University of Porto, Rua Dr. Roberto Frias, 4200-465 Porto, Portugal*

*2. ALiCE - Associate Laboratory in Chemical Engineering, Faculty of Engineering, University of Porto, Rua Dr. Roberto Frias, 4200-465 Porto, Portugal*

Number of pages: 3

Number of figures: 5

*Corresponding author. Tel.: +351 920427795; fax: +351 225081449

*E-mail address:* ivanou@fe.up.pt (D. Ivanou); mendes@fe.up.pt (A. Mendes)

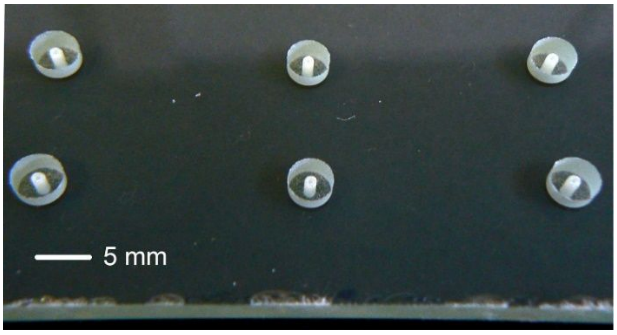


**Figure S1**. Photograph the cover glass for M-DSSCs with electrolyte injection holes made by laser ablation.

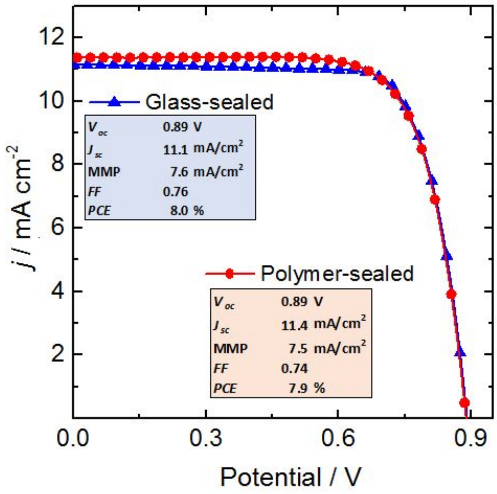


**Figure S2**. Typical photocurrent *vs*. applied potential curves obtained under simulated AM1.5G (100 mW·cm$^{-2}$) illumination for cobalt-mediated M-DSSCs conventionally sealed with Surlyn thermoplastic (circles) and sealed using glass frit (triangles).

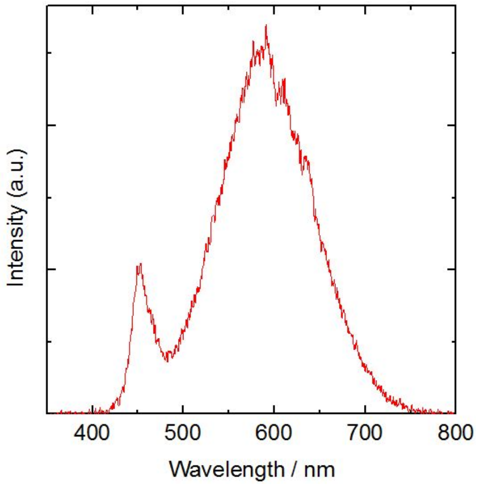


**Figure S3**. Emission spectra of white LED lamp (Color temperature 2700 K) used for *J-V* characterization of the M-DSSCs under artificial room light.

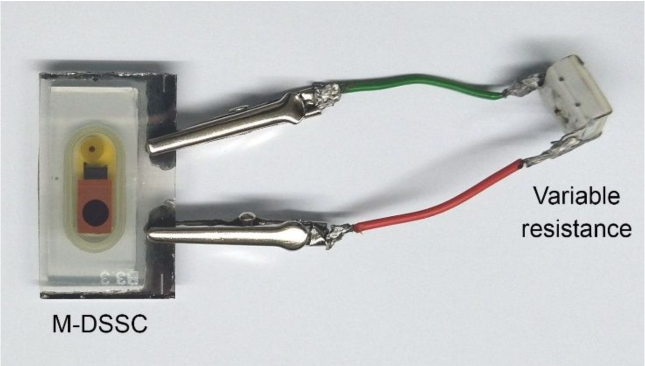


**Figure S4**. Photograph of M-DSSC device connected to a variable resistance during light-soaking tests to simulate the device operation at the maximum power point.

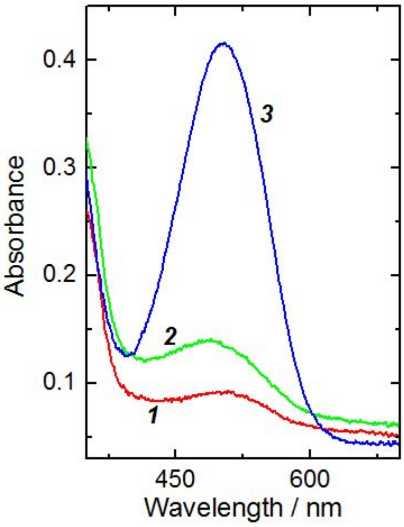


**Figure S5**. Absorption spectra of acetonitrile after immersion of the carbon counter-electrode for 10 minutes at 65 $^{o}$C (***1***) and 75 $^{o}$C (***2***). Absorbance spectra of 0.01 M solution of Y123 dye (***3***) in acetonitrile/*t*-butanol mixture (1:1 volume ratio).
